# Key aims of the special series “the meaning of behavioral medicine in the psychosomatic field”

**DOI:** 10.1186/s13030-016-0055-7

**Published:** 2016-02-24

**Authors:** Mutsuhiro Nakao

**Affiliations:** Teikyo University Graduate School of Public Health & Department of Psychosomatic Medicine, Teikyo University Hospital, 2-11-1, Kaga, Itabashi, Tokyo, 173-8605 Japan

The practice of psychosomatic medicine is closely related to that of behavioral medicine, particularly in terms of the bio-psycho-social aspect of health, as has been demonstrated in the previous articles of BioPsychoSocial Medicine [1–3]. Behavioral medicine is an interdisciplinary field combining medicine, psychology and social science, and the integration of behavioral medicine with psychosomatic medicine would contribute to the understanding of psychosomatic phenomena in terms of treatment and the prevention of disease [4]. Such integration has been gradually achieved in fields of research and clinical practice during recent 50 years, but not yet satisfactory in terms of research and clinical practice.

For example, among the major psychosomatic medicine journals, the percentage of articles focusing on behavioral medicine [MeSH] was estimated by a PubMed search in September 2015, and the results were as follows; 4.5 % (=212/4706) for “Psychosomatic Medicine”, 3.7 % (=164/4388) for “Psychosomatics”, 2.9 % (=129/4488) for “Journal of Psychosomatic Research”, and 2.2 % (=60/2768) for =2.2 %. It was 8.8 % (=16/181) for this journal, “BioPsychoSocial Medicine”, which was launched in 2007.

In the same way, the percentage of articles focusing on psychosomatic OR mind/body [text word] was estimated for the major behavioral medicine journals, and the results were as follows; 2.3 % (=12/531) for “Behavioral Medicine”, 1.3 % (=11/856) for “International Journal of Behavioral Medicine”, and 0.7 % (=12/1626) for Journal of Behavioral Medicine. The percentages were smaller than those of the psychosomatic medicine journals. These figures mean that it is necessary for specialists engaging in psychosomatic medicine not only to sharpen their own research into behavioral medicine but also to broaden their findings toward specialists in behavioral medicine. Psychosomatic medicine encompasses all aspects of the interrelationships between the biological, psychological, social, and behavioral factors of health and illness, and is not limited to mind and body connections in humans. Thus, it is meaningful that behavioral and social aspects of health and illness are discussed in the current issue of this journal [1].

In Japan, a total of 80 medical schools exist, and curriculums for medical students have been changed in accordance with international standards. In the revised curriculums, aspects of behavioral and social sciences must be included and it is a great chance to facilitate behavioral medicine. In these circumstances, the Japanese Society of Behavioral Medicine established a working group to develop a core curriculum for behavioral medicine and has discussed the clinical, psychological, and public health aspects of behavioral medicine. Specifically, two purposes are intended in this special series. The first is to summarize the behavioral and social aspects of health and illness in the psychosomatic filed in order to achieve full cooperation among different specialists in terms of prevention and treatment of psychosomatic illnesses as well as promotion of mind/body health. The second is to emphasize the importance of education on behavioral medicine, exemplified by the establishment of a core curriculum for behavioral medicine in Japanese medical schools.
